# Can a New Scoring System Improve Prediction of Pulmonary Hypertension in Newly Recognised Interstitial Lung Diseases?

**DOI:** 10.1007/s00408-020-00346-1

**Published:** 2020-03-23

**Authors:** Małgorzata Sobiecka, Katarzyna Lewandowska, Jarosław Kober, Monika Franczuk, Agnieszka Skoczylas, Witold Tomkowski, Jan Kuś, Monika Szturmowicz

**Affiliations:** 1 1st Department of Lung Diseases, National Tuberculosis and Lung Diseases Research Institute, Plocka 26, 01-138 Warsaw, Poland; 2 Department of Radiology, National Tuberculosis and Lung Diseases Research Institute, Warsaw, Poland; 3 Department of Respiratory Physiopathology, National Tuberculosis and Lung Diseases Research Institute, Warsaw, Poland; 4 Department of Geriatrics, National Institute of Geriatrics, Rheumatology and Rehabilitation, Warsaw, Poland

**Keywords:** Interstitial lung disease, Pulmonary hypertension, Total lung capacity, Diffusion capacity for carbon monoxide, NT-proBNP

## Abstract

**Introduction:**

Pulmonary hypertension (PH) is a well-recognised complication of interstitial lung diseases (ILD), which worsens prognosis and impairs exercise capacity. Echocardiography is the most widely used, non-invasive method for PH assessment. The aim of our study was to identify the factors predictive for echocardiographic signs of PH in newly recognised ILD patients.

**Methods:**

Ninety-three consecutive patients (28F/65M) with different ILD were prospectively evaluated from January 2009 to March 2014. Pulmonary function testing, 6-min walk distance (6MWD), initial and sixth minute room air oxygen saturation, NT-proBNP and echocardiography were assessed in each patient. Echocardiographic PH probability was determined according to the 2009 ESC/ERS guidelines.

**Results:**

In 41 patients (Group B) increased PH possibility has been diagnosed on echocardiography, in 52 patients (Group A)—low PH probability. Most pronounced differences (*p* ≤ 0.0005) between groups B and A concerned: age, 6MWD, room air oxygen saturation at 6 min, DLCO and TLC/DLCO index (57.6 vs 43.8 years; 478 vs 583 m; 89.1% vs 93.4%; 54.8% predicted vs 70.5% predicted and 1.86 vs 1.44; respectively). Univariate analysis showed four-fold increased probability of PH when TLC/DLCO exceeded 1.67. A scoring system incorporating age, TLC/DLCO index, 6MWD and room air oxygen saturation at 6 min provided high diagnostic utility, AUC 0.867 (95% CI 0.792–0.867).

**Conclusion:**

ILD patients with TLC/DLCO index > 1.67 have a high likelihood of PH and should undergo further evaluation. The composite model of PH prediction, including age, 6-min walk test and TLC/DLCO was highly specific for recognition of PH on echocardiography.

## Introduction

Interstitial lung disease (ILD) encompasses a large heterogeneous group of inflammatory and fibrotic lung diseases. Chronic fibrosing ILD, such as idiopathic pulmonary fibrosis (IPF), chronic hypersensitivity pneumonitis, nonspecific interstitial pneumonia (NSIP), and sarcoidosis are the most common entities in this group of disorders [1, 2]. Pulmonary hypertension (PH) is a well-recognised complication of several ILDs, worsening prognosis and impairing exercise capacity [3–8]. Among patients with ILD, PH has been most often studied in IPF, sarcoidosis and hypersensitivity pneumonitis [9–11]. The prevalence of PH ranges from 8 up to 86% in patients with IPF, and from 6 up to 74% in patients with sarcoidosis—depending on the diagnostic algorithm, the PH definition, the stage and severity of the underlying disease [4, 12–15].

Despite increasing awareness of PH, diagnosis is often delayed due to common symptoms of PH and ILD, such as shortness of breath and exercise limitation [16]. Furthermore, PH may also occur in non-hypoxemic patients with mild-to-moderate functional impairment and in sarcoidosis—in patients without evident features of interstitial lung disease [5, 17]. As the presence of PH is the known marker of poor prognosis in ILD, it seems necessary to stratify patients and identify those who need assessment for PH. Moreover, early recognition of PH in patients with ILD may be important, in terms of planning diagnostic tests (higher risk of bleeding during lung biopsies in case of PH), establishing an indication for long-term oxygen therapy in hypoxemic patients and considering qualification for lung transplantation [18].

Right heart catheterisation is the gold standard for the diagnostic confirmation of PH. Nevertheless, it is not recommended as a routine screening tool in patients with lung diseases because it is an invasive procedure [19]. Right heart catheterisation is currently recommended for the diagnosis of group 3 PH only in selected circumstances, such as listing for lung transplantation, suspicion of pulmonary arterial hypertension or chronic thromboembolic PH and considering PH-directed therapy in randomised clinical trials [20].

Echocardiography is the most widely used and recommended non-invasive method for PH assessment [20]. Screening for PH with echocardiography should be applied in patients with clinical data suggestive of increased risk of this complication. Unfortunately, the optimal method of qualification of ILD patients for echocardiography has not been established.

Some authors suggest that elevated forced vital capacity to diffusing lung capacity for carbon monoxide (FVC/DLCO) ratio may be a useful, non-invasive prognostic marker for PH in patients with IPF and systemic sclerosis [21–23]. However, there are various situations that may alter the FVC (e.g. emphysema combined with lung fibrosis, obstructive disorders), and the utility of measuring this ratio as a screening tool might be reduced [24]. The assessment of total lung capacity (TLC) is the gold standard for detecting a restrictive pattern, and it is a part of routine diagnostic work-up of patients with ILD, as well as patients with PH reported to international registers [25]. It was found that only about 60% of those with a restrictive spirometric pattern defined as FVC < 80% predicted and FEV_1_/FVC > 0.7 before bronchodilatation, have a true restrictive impairment of lung function defined as a TLC below the lower limit of normal [26]. In addition, TLC has been shown to be significantly associated with mortality in patients with group 3 PH [27] and in an unselected population of elderly patients [28].

Therefore, the aim of the present prospective study was to assess whether a new non-invasive ratio, plethysmographic TLC% predicted divided by corrected for haemoglobin DLCO% predicted (TLC/DLCO) can provide useful information for screening PH in patients with newly recognised ILD and to create the scoring system for prediction of PH assessed by echocardiography.

## Materials and Methods

### Patient Population

All consecutive patients presented to a single pulmonary department between 2009 and 2014, diagnosed with various ILD, according to the current at that time guidelines [29–31], entered the study.

The exclusion criteria were: age < 18 years, significant left heart disease, acute pulmonary embolism or chronic thromboembolic PH, severe renal or liver insufficiency and the presence of severe comorbidities with poor prognosis (e.g. cancer, severe neurological disease). The patients with a known diagnosis of connective tissue disease-ILD were also excluded due to possibility of pulmonary arterial hypertension in this group.

### Procedures

2D-Doppler echocardiographic transthoracic examination was performed with Siemens Accusuin, Sequoia, and Toshiba Medical Systems SSH-880 CV/W1 Artida, as a part of the initial evaluation. Arbitrary criteria of PH were adopted according to the 2009 ESC/ERS guidelines (Table 1) [32].

**Table 1:** Arbitrary echocardiographic criteria for estimating the presence of pulmonary hypertension (PH) according to ERS/ESC 2009 [32]

TRV max	PASP	Additional echocardiographic variables suggestive of PH*	Echocardiographic probability of PH
≤ 2.8 m/s	≤ 36 mmHg	Absent	Unlikely
≤ 2.8 m/s	≤ 36 mmHg	Present	
2.9–3.4 m/s	37–50 mmHg	Absent or present	Possible
> 3.4 m/s	> 50 mmHg	Absent or present	Likely

*PASP* pulmonary arterial systolic pressure, *PH* pulmonary hypertension, *TRV* tricuspid regurgitant velocity

*Increased velocity of pulmonary valve regurgitation, short right ventricular outflow acceleration time, increased right heart chambers, increased right ventricular wall thickness, flattening or paradoxical movement of interventricular septum

Computed tomography of the chest was performed with Somaton Sensation 16.

Exercise capacity was assessed with a 6 min walk test (6MWT) performed on a corridor in accordance with ATS guidelines [33]. Distance covered during 6 min of walking (6MWD), as well as baseline (sat 0) and sixth minute (sat 6) room air oxygen saturation were noted.

All pulmonary function measurements were performed with the use of an integrated measuring device MasterScreen Body/Diffusion by Jaeger (Germany 2002), following the ERS/ATS recommendations [34]. The values of pulmonary function indices were reported as a percentage of the predicted values according to ERS reference equations [35], and Falaschetti [36] for FEV_1_ and FVC values. The measurements of diffusing lung capacity for carbon monoxide (DLCO) were performed by the single breath method, with helium gas as a marker, according to ERS standards [37]. The results were presented as a percentage of the predicted values with correction for haemoglobin.

Serum N-terminal pro-B-type natriuretic peptide (NT-proBNP) in venous blood samples was analysed at the hospital’s accredited laboratory, using a standard procedure (Elecsys proBNP II, Cobas e411, Roche Diagnostics GmbH, Germany). The upper limit of normal range was 125 pg/ml.

### Statistical Analysis

All analyses were performed with R-a software environment for statistical computing and graphics (https://www.r-project.org/) [38]. Continuous variables were presented as means and standard deviations, and categorical ones—as percentages of the entire population studied. Distributions’ normality and homogeneity of variance of continuous variables in groups with different PH probability were checked with Shapiro–Wilk test and *F* test, respectively. If both criteria were fulfilled, *T*-Student test and otherwise *U* Mann–Whitney test was used. Categorical variables distribution was compared with Pearson’s test with its modifications if applicable. Youden method was used for calculation of cut off values of parameters with the highest specificity and sensitivity for PH prediction. Regression analysis (odds ratios and 95% confidence intervals) was applied to assess the PH risk combined with different factors.

## Results

### Baseline Characteristics

The examined group consisted of 93 patients, 65 males, 28 females, mean age 49.9 ± 13.9 years. Sarcoidosis was diagnosed in 42 patients (stage I—in 15, II—in 21, III—in 3 and IV—in 3), hypersensitivity pneumonitis—in 22, IPF—in 21, and other types of idiopathic interstitial pneumonia—in 8 patients.

The results of echocardiography revealed a low probability of PH (PH unlikely) in 52 patients, intermediate probability (PH possible) – in 38, and high probability (PH likely)—in 3. For further analysis, two echocardiographic categories have been defined: group A—low probability of PH, 52 patients (56%), and group B—increased probability of PH (PH possible and PH likely), 41 patients (44%). Mean SPAP has been calculated as 29.2 ± 3.05 in group A and 38.34 ± 7.22 in group B (*p* = 0.00000001).

Mean age, pulmonary function test values, 6MWT parameters and NT-proBNP concentration in both groups are presented in Table 2.

**Table 2:** Comparison of chosen parameters (means ± SD) in the groups with different probability of PH on echocardiography

Parameter	Group A (low PH probability)	Group B (increased PH probability)	*p*
Age (years)	43.8 ± 12.2	57.6 ± 12.1	0.0000006
Smoking (pack-years)	6.9 ± 14.0	17.8 ± 21.8	0.0043
6MWD (m)	583.1 ± 111.6	478.2 ± 109	0.0000204
Sat 0 (%)	96.6 ± 2.3	95.8 ± 1.9	0.00227
Sat 6 (%)	93.4 ± 5.2	89.1 ± 7.0	0.000566
Desaturation (%)	3.3 ± 3.9	6.7 ± 5.7	0.0013
FEV1%FVC	78.5 ± 6.3	75.8 ± 9.3	0.1746
FVC (% pred)	94.5 ± 22.7	95.0 ± 21.3	0.9126
TLC (% pred)	96.0 ± 19.4	91.9 ± 21.9	0.3524
DLCO (% pred)	70.5 ± 20.8	54.8 ± 22.2	0.0005
TLC/DLCO	1.44 ± 0.6	1.86 ± 0.6	0.00001
FVC/DLCO	1.4 ± 0.45	1.82 ± 0.67	0.00001
VC/DLCO	1.43 ± 0.48	1.94 ± 0.68	0.00001
NT-proBNP (pg/ml)	58.4 ± 53.9	150.7 ± 300.7	0.019

*DLCO* diffusion lung capacity for carbon monoxide, *FEV*_1_ forced expiratory volume in one second, *FVC* forced vital capacity, *6MWD* 6-min walk distance, *NT-proBNP* N-terminal brain natriuretic peptide, *PH* pulmonary hypertension, *Sat0* baseline room air oxygen saturation, *Sat6* sixth minute room air oxygen saturation, *TLC* total lung capacity, *TLC/DLCO* total lung capacity% predicted/diffusion lung capacity for carbon monoxide corrected for haemoglobin % predicted, *FVC/DLCO* forced vital capacity %predicted/diffusion lung capacity for carbon monoxide corrected for haemoglobin % predicted, *VC/DLCO* vital capacity %predicted/diffusion lung capacity for carbon monoxide corrected for haemoglobin % predicted

Patients belonging to group B were significantly older compared to those from group A (mean age 57.6 vs 43.8 years, respectively). They covered a significantly shorter distance during 6MWT (478 vs 583 m), with significantly lower mean sat 0 (95.8 vs 96.6%) and mean sat 6 (89.1 vs 93.4%) compared to group A. Plethysmography revealed a tendency towards lower TLC in group B compared to those of group A (92 vs 96% pred.), in spirometry, FVC and FEV_1_%FVC were comparable in both groups. Mean DLCO value was significantly lower in group B compared to group A, (54.8 vs 70.5% pred.; *p* = 0.0005). The average TLC/DLCO index was 1.86 in group B and 1.44 in group A, (*p* = 0.00001).

### ROC Analysis

ROC analysis revealed the highest value of age, 6MWD, sat 6, and TLC/DLCO index, for PH prediction (Table 3). Optimal specificity and sensitivity were calculated for subsequent cut off values: age > 53 years, 6MWD < 507.5 m, sat 6 < 93%, and TLC/DLCO ratio > 1.67 (Table 4).

**Table 3:** PH prediction: diagnostic utility of various parameters (ROC analysis)

Parameter	AUC	95% CI	*p*
Age (years)	0.803	0.707–0.803	0.000033
Pack-years	0.665	0.555–0.665	0.03
6MWD (m)	0.761	0.663–0.761	0.0002
Sat 0 (%)	0.683	0.570–0.680	0.02
Sat 6 (%)	0.710	0.600–0.710	0.005
Desaturation (%)	0.696	0.585–0.696	0.01
DLCO (% pred)	0.710	0.602–0.713	0.006
TLC/DLCO	0.770	0.660–0.770	0.0004
FVC/DLCO	0.77	0.675–0.777	0.0069
VC/DLCO	0.78	0.666–0.788	0.0053
NT-proBNP (pg/ml)	0.645	0.528–0.645	0.07

*AUC* area under curve, *DLCO* diffusion lung capacity for carbon monoxide, *6MWD* 6-min walk distance, *NT-proBNP* N-terminal brain natriuretic peptide, *PH* pulmonary hypertension, *Sat0* baseline room air oxygen saturation, *Sat6* sixth minute room air oxygen saturation, *TLC/DLCO* total lung capacity% predicted/diffusion lung capacity for carbon monoxide corrected for haemoglobin % predicted, *FVC/DLCO* forced vital capacity %predicted/diffusion lung capacity for carbon monoxide corrected for haemoglobin % predicted, *VC/DLCO* vital capacity %predicted/diffusion lung capacity for carbon monoxide corrected for haemoglobin % predicted

**Table 4:** Optimal cut off values of different parameters for PH prediction

Parameter	Cut off	Specificity	Sensitivity	PPV	NPV
Age (years)	53	0.85	0.76	0.79	0.81
Pack-years	1.75	0.69	0.61	0.58	0.71
6MWD (m)	507.5	0.76	0.63	0.68	0.72
Sat 0 (%)	96	0.76	0.68	0.69	0.75
Sat 6 (%)	93	0.75	0.65	0.67	0.73
Desaturation (%)	4	0.71	0.65	0.63	0.72
DLCO (% pred)	68	0.67	0.78	0.65	0.79
TLC/DLCO	1.67	0.92	0.65	0.87	0.77
NT-proBNP (pg/ml)	38	0.49	0.77	0.54	0.74

*DLCO* diffusion lung capacity for carbon monoxide, *6MWD* 6-min walk distance, *NT-proBNP* N-terminal brain natriuretic peptide, *PH* pulmonary hypertension, *Sat0 *baseline room air oxygen saturation, *Sat6* sixth minute room air oxygen saturation, *TLC/DLCO* total lung capacity% predicted/diffusion lung capacity for carbon monoxide corrected for haemoglobin % predicted

**Fig. 1:**
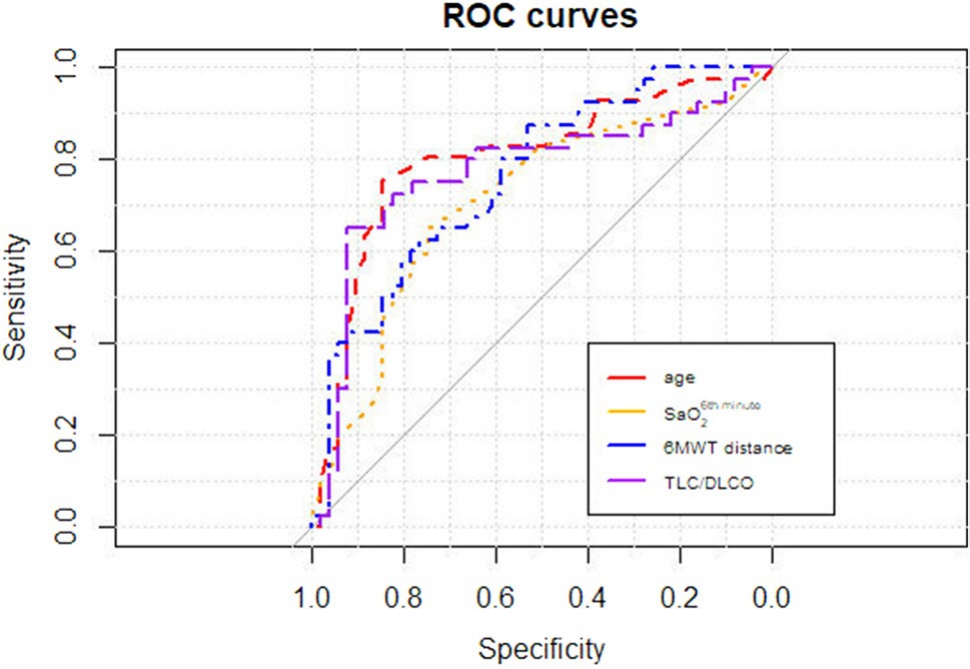
Diagnostic value of various parameters for PH prediction (ROC curves)

**Fig. 2:**
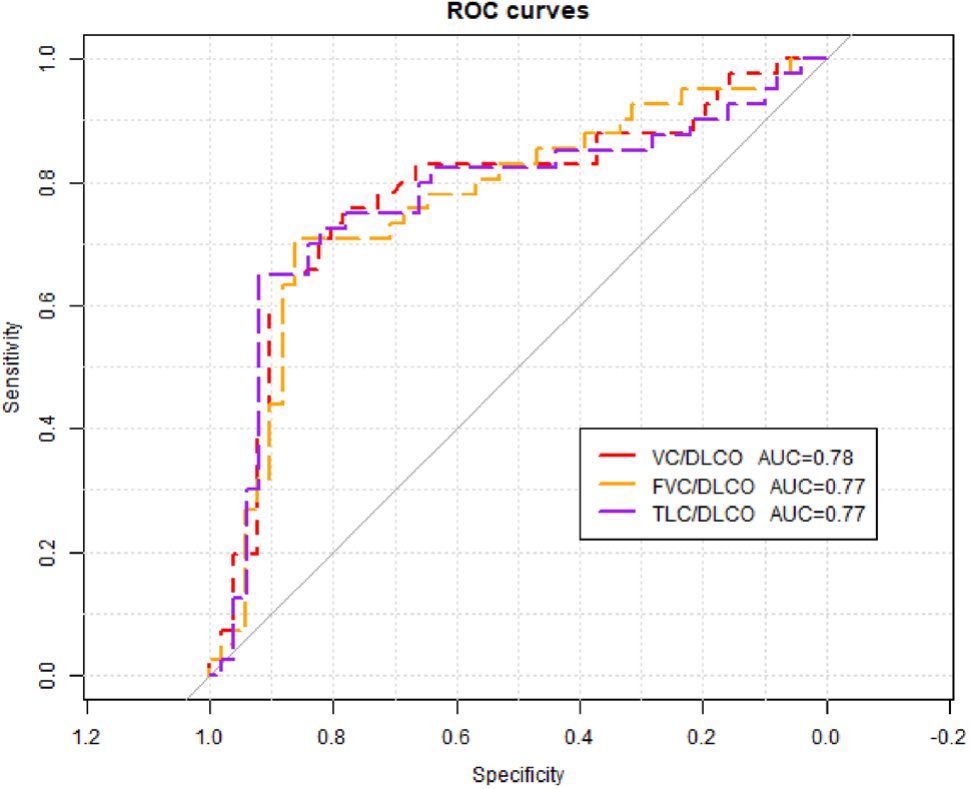
Comparison of diagnostic values of VC/DLCO, FVC/DLCO and TLC/DLCO for PH prediction (ROC curves)

The ROC curves illustrating the diagnostic utility of age, 6MWD, sat 6 and TLC/DLCO ratio for PH prediction are shown on Fig. 1. The comparison of VC/DLCO, FVC/DLCO and TLC/DLCO are shown on Fig. 2.

Univariate analysis revealed that the risk of PH on echocardiography was increased by four times in the patients with TLC/DLCO exceeding 1.67 (Table 5).

**Table 5:** Risk of PH—univariate analysis

Parameter	Cut off	OR	95% CI	*p*
Age (years)	53	1.093	1.049–1.138	0.000019
Pack-years	1.75	1.036	1.008–1.065	0.01
6MWD (m)	507.6	0.991	0.987–0.996	0.00016
Sat 6 (%)	93	0.888	0.821–0.961	0.003
Desaturation (%)	4	1.162	1.052–1.284	0.0032
DLCO (%predicted)	68	0.967	0.947–0.987	0.0015
TLC/DLCO	1.67	4.393	1.557–12.394	0.0052
NT-proBNP (pg/ml)	38	1.008	1.001–1.015	0.0259

*DLCO* diffusion lung capacity for carbon monoxide, *6MWD* 6-min walk distance, *NT-proBNP* N-terminal brain natriuretic peptide, *PH* pulmonary hypertension, *Sat* 6 sixth minute room air oxygen saturation, *TLC/DLCO*: total lung capacity% predicted/diffusion lung capacity for carbon monoxide corrected for haemoglobin % predicted

### PH—Prediction Scoring Model

**Fig. 3:**
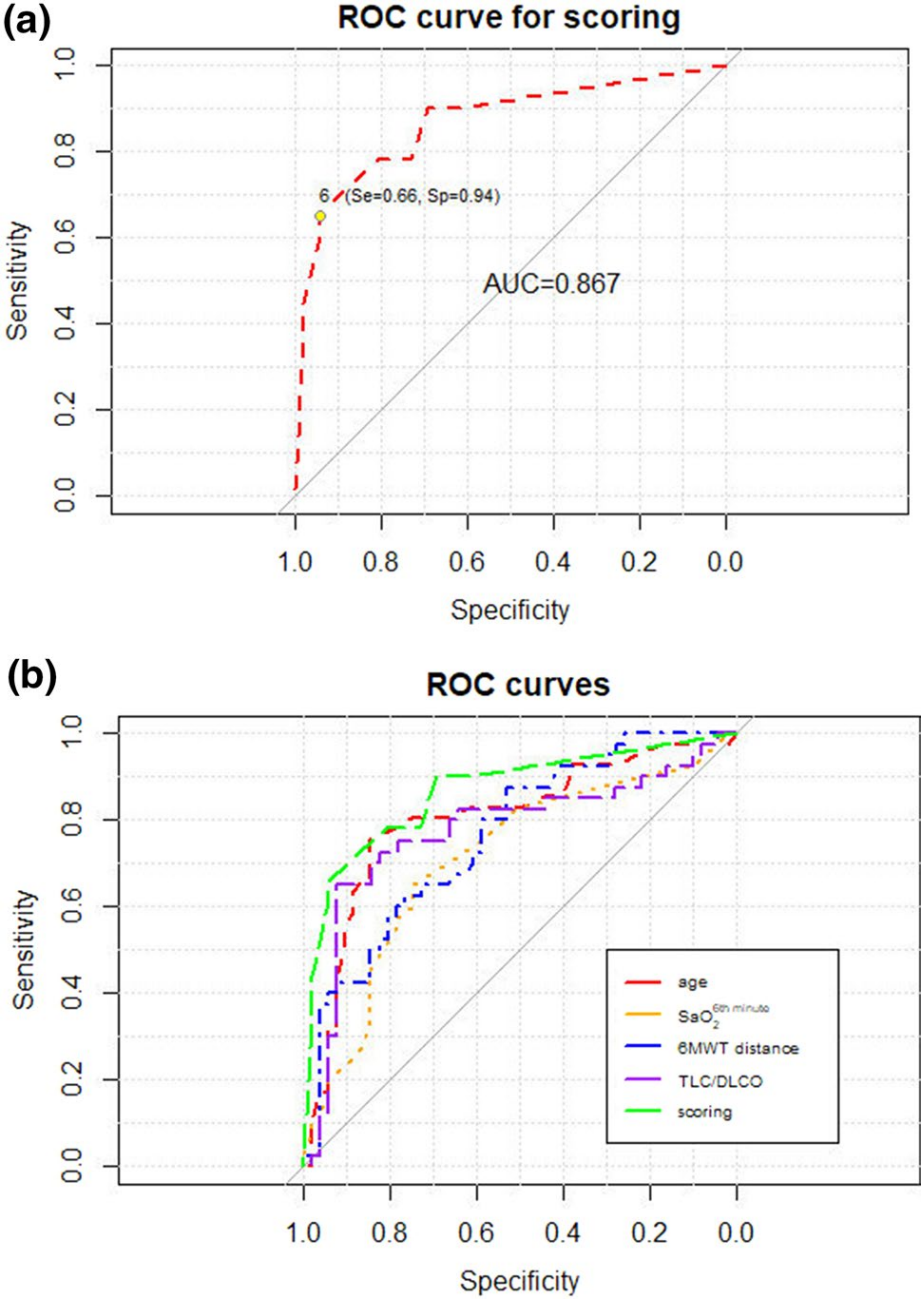
**a** and **b** Diagnostic value of composite model for PH prediction: **a**—as a single parameter, **b**—in comparison with other predictive factors (ROC curves)

A scoring system for prediction of PH assessed by echocardiography has been created. The parameters exceeding optimal cut off values have been scored according to the results of ROC analysis: age > 53 years and TLC/DLCO > 1.67, three points each, 6MWD < 507.5 m and sat 6 < 93%—two points each (maximal score—10 points). The AUC of the scoring model for PH prediction was 0.867 (95% CI 0.792–0.867). Optimal cut off score, indicating the increased probability of PH was 6 points, the specificity, sensitivity, positive predictive value and negative predictive values were: 94%, 66%, 90% and 78%, respectively. ROC curves for PH prediction according to scoring results are shown on Fig. 3a and b.

## Discussion

This is the first report on TLC/DLCO ratio as a predictive marker for PH in newly diagnosed patients with various non-connective tissue disease-ILD. Moreover, a simple scoring system consisting of age, TLC/DLCO ratio, 6-min room air oxygen saturation and 6MWD was highly specific for the recognition of PH on echocardiography, with PPV of 90%.

The most substantial difference between the groups with low and increased probability of PH in our study concerned DLCO (mean predicted values were 70.5% and 54.8%, respectively, *p* = 0.0005). In patients with the increased probability of PH we observed the disproportionate decrease of DLCO compared to TLC, indicating the possibility of vascular lung disease. This has resulted in significantly higher TLC/DLCO index in group B, compared to group A (1.86 and 1.44, respectively, *p* = 0.00001). Univariate analysis revealed the four-fold increase of PH probability in the patients with TLC/DLCO ratio exceeding 1.67. TLC/DLCO > 1.67 was also the most specific indicator of PH diagnosed by echocardiography.

The ability to predict PH due to ILD was documented both in sarcoidosis [39, 40] and in IPF patients with decreased DLCO [3, 21, 22, 41]. Lettieri et al. found that DLCO < 40% pred. in the patients with end stage IPF, indicated PH on right heart catheterisation with 94% specificity and 65% sensitivity [3]. In our study, DLCO < 68% pred. appeared significant for PH prediction, because the examined group consisted of patients with newly diagnosed ILD, with less severe lung function disturbances.

The other authors applied FVC/DLCO index for PH prediction in patients with IPF [21, 22]. High diagnostic value of combined FVC/DLCO index and resting room air pulse oximetry has been documented by Zisman et al. [22] and more recently Alkukhun et al. [21]. Therefore, we compared diagnostic value of three indexes: FVC/DLCO, VC/DLCO and TLC/DLCO for PH prediction in the present study group. All the three indexes had comparable diagnostic utility. We decided to use TLC/DLCO ratio as the assessment of TLC is the best test to correctly differentiate restrictive from normal lung function. The reduction of TLC is a stronger risk factor for mortality than FVC in an unselected population of elderly patients [28], and it was associated with mortality among patients with group 3 PH [27].

6MWT results showed significantly longer distance in group A compared to group B (583 vs 478 m, respectively). In patients with PH due to lung diseases, 6MWD is influenced by severity of lung disease, and also by comorbidities and age. Initial and end-walking oxygen saturation during 6MWT may be more valuable PH predictors than walking distance, especially in ILD patients. Mean sixth minute oxygen saturation was significantly lower in group B compared to group A (89.1% and 93.4%, respectively). The optimal sixth minute oxygen saturation cut off was 93% (75% specificity, 65% sensitivity). The published data on this subject are contradictory. Lettieri et al. confirmed significantly lower end-walk oxygen saturation in patients with PH compared to those without PH (80% and 88%, respectively), in end stage IPF patients [3], but Modrykamien et al. did not find the utility of sixth minute oxygen saturation and 6MWD for PH prediction in IPF [42].

In our group of patients, the mean NT-proBNP serum level was significantly higher in group B compared to group A (151 pg/ml vs 58 pg/ml). Nevertheless, in most patients, NT-proBNP concentrations were within normal limits. Therefore, we conclude that the clinical utility of NT-proBNP for PH prediction in newly diagnosed ILD is low. It seems that low NT-pro BNP at diagnosis may be used as a factor with negative predictive value. Andersen et al. found the concentration < 95 ng/l excluding PH on echocardiography in patients with ILD [43].

The composite model of PH prediction, created by our group, consisted of the best single predictors, such as age, TLC/DLCO ratio, sixth minute room air oxygen saturation and 6MWD. The score of 6 points was highly specific for the recognition of PH on echocardiography (94%), with PPV of 90%. This is, to our knowledge, the first attempt to create universal scoring system for PH prediction in various ILDs.

The other model of clinical PH prediction has been presented by Thakhar in scleroderma patients [44]. It was composed of NT-proBNP ≥ 209.8 and/or DLCO < 70.3% predicted and FVC/DLCO ≥ 1.82 (100% sensitivity and 77.8% specificity). Recently, another model of PH prediction was described by Furukawa et al. in IPF patients [41]. This model included DLCO < 50%, index of pulmonary artery/descending aorta diameters > 0.9 and PaO_2_ < 80 mmHg.

There are some limitations of our study. Firstly, as a single tertiary centre study, the ability to avoid data bias was limited. Secondly, the outcome might be influenced by the relatively small number of patients and the heterogeneity of the study population comprised patients with sarcoidosis, hypersensitivity pneumonitis, IPF, and other idiopathic interstitial pneumonias. On the other hand, the included patient population reflects cases encountered in “real-life” clinical practice, and our study aimed to identify easily ascertainable characteristics that would predict the echocardiographic confirmation of PH in newly diagnosed ILD patients. Lastly, our study is limited by the lack of confirmatory right heart catheterization. We used the hemodynamic data obtained by Doppler echocardiography—a noninvasive, inexpensive and widely available method for assessment of PH. Right heart catheterization is not used as a routine screening tool in patients with lung diseases due to its invasiveness, and this method is currently recommended for the diagnosis of group 3 PH, solely in selected circumstances [45].

The authors are aware that the applied scoring model for clinical PH probability assessment needs validation. Nevertheless, the preliminary results are auspicious, and worth considering as clinically useful and reliable.

## Conclusion

A simple scoring system comprised age, TLC/DLCO ratio and walking test results (air room oxygen saturation at sixth minute and 6MWD) was highly specific for the recognition of PH on echocardiography. Moreover, TLC/DLCO ratio alone exceeding 1.67 increased the probability of PH by four times. This index was also the most specific indicator for PH diagnosed on echocardiography in patients with ILD and should undergo further evaluation.

## Data Availability

The datasets collected and analysed during the current study are available from the corresponding author on reasonable request.
